# Sensor Anomaly Detection in Wireless Sensor Networks for Healthcare

**DOI:** 10.3390/s150408764

**Published:** 2015-04-15

**Authors:** Shah Ahsanul Haque, Mustafizur Rahman, Syed Mahfuzul Aziz

**Affiliations:** 1 School of Engineering, University of South Australia, Mawson Lakes, SA 5095, Australia; 2 Department of Defence, Defence Science and Technology Organization, SA 5111, Australia; E-Mail: mustafizur.rahman@dsto.defence.gov.au; 3 School of Engineering, University of South Australia, Mawson Lakes, SA 5095, Australia; E-Mail: mahfuz.aziz@unisa.edu.au

**Keywords:** wireless sensor networks, healthcare, medical sensors, sensor fault, sensor anomaly detection, prediction

## Abstract

Wireless Sensor Networks (WSN) are vulnerable to various sensor faults and faulty measurements. This vulnerability hinders efficient and timely response in various WSN applications, such as healthcare. For example, faulty measurements can create false alarms which may require unnecessary intervention from healthcare personnel. Therefore, an approach to differentiate between real medical conditions and false alarms will improve remote patient monitoring systems and quality of healthcare service afforded by WSN. In this paper, a novel approach is proposed to detect sensor anomaly by analyzing collected physiological data from medical sensors. The objective of this method is to effectively distinguish false alarms from true alarms. It predicts a sensor value from historic values and compares it with the actual sensed value for a particular instance. The difference is compared against a threshold value, which is dynamically adjusted, to ascertain whether the sensor value is anomalous. The proposed approach has been applied to real healthcare datasets and compared with existing approaches. Experimental results demonstrate the effectiveness of the proposed system, providing high Detection Rate (DR) and low False Positive Rate (FPR).

## 1. Introduction

Wireless Sensor Networks (WSNs) are used in numerous application domains. WSNs are networks of distributed wireless sensors with energy and processing constraints. Their use is perceived to be limited to low data intensive applications. Recent advances in low power hardware architecture and communication protocols [1,2,3,4,5] have demonstrated the use of WSNs even in high data intensive applications, for example, visual sensing and image communication. Such advances in wireless sensing and networking technologies for diverse applications are likely to be key enablers for the effective integration of physical and cyber worlds, a precondition for the successful development of the Internet of Things (IoT). Wireless Sensor Networks can be used in the medical domain to enhance the provisioning and management of healthcare services [6]. Wireless medical sensors are small, resource constrained devices and capable of collecting various physiological parameters, such as Heart Rate (HR), Pulse, Oxygen Saturation (SpO_2_), Respiration and Blood Pressure (BP). These sensors are usually battery operated, attached to the subject’s body and are continuously monitored in hospital or home environments [7]. Also there are non-intrusive sensors [8] capable of analyzing physiological conditions and detect falls. These sensed data provide valuable information for doctors, nurses and caregivers to determine the medical condition of the subject. As in-hospital monitoring of subjects for long periods of time is costly, a viable option is to keep the non-emergency subjects in their home and continue monitoring using remote medical sensors [9]. Medical sensors with wireless transmission capability, such as MICAz [10], TelosB [11], Shimmer [12] and IRIS [13] provide flexibility for the subjects in terms of mobility and movement [14]. As the caregiver may not be present all the time to monitor the sensed data, it is important to ensure the accuracy and reliability of the data to raise an alarm in case of emergency.

Collected sensor data may be inaccurate due to sensor fault and resource constraint of the sensor node such as limitation of power and transmission capability [15]. Other factors of data inaccuracy may include sensor displacement, transmission interference, and malicious data injection. The sensor data may also be unreliable due to transmission error, all of which may result generating false alarms. False alarms have negative impact on healthcare system, for example, causing fatigue to the caregivers, which may lead to degradation of the quality of service and waste of valuable time and money. It is important to detect data inaccuracies at sensor nodes because the result of collecting faulty data and injecting it to the system may compromise the system and generate many false alarms. This may lead to undesirable consequences as the fatigued caregivers may end up attending false alarms when real emergency medical case may be left unattended.

In case of continuous monitoring, the amount of accumulated data grows over time. Therefore, in the absence of fast processing and alarm generation systems, the timely detection of emergency medical conditions may not be possible. Therefore a real-time, fast and reliable system to detect unreliable or faulty sensor data, and then identify and isolate potential false alarms, and finally generate true alarms can improve the quality of care. Various sensor anomaly detection systems have been proposed and applied to date [16,17,18,19]. Distributed techniques [20] measures the dissimilarity of sensor observations in principal component space and can detect anomalous data at specific sensor level, however the resource requirement is not sufficient for battery operated wireless sensors. On the other hand, the centralized approach [21] is not an energy efficient way of routing in WSN and can deplete the sensors’ energy very quickly as all data need to be transmitted to the sink for processing.

In this paper, we propose a novel approach to detect sensor anomaly and reduce false alarms by developing prediction based methods to compare and detect anomalies. Although traditional anomaly detection methods [22] can detect and exclude anomalies from the data, however, anomalous values in healthcare are important as anomalies may result from a true medical condition. Therefore, it is important to adequately analyze anomalies to determine whether the anomalous values are indeed faulty or if they represent true medical conditions. Based on this analysis, the decision to generate true alarm or false alarm is made. Proposed anomaly detection method utilizes the spatio-temporal correlation that exists among physiological parameters. The data collected from various sensor nodes are transmitted to the base station or to nodes with higher processing and memory capacity, and a prediction model is generated based on the historic data. Then a dynamic threshold based error computation is performed followed by majority voting analysis to identify the sensor anomaly and generate alarms. The proposed approach has been applied to real healthcare datasets and compared with other related approaches. Experimental results show the effectiveness of the proposed approach, providing high Detection Rate (DR) and low False Positive Rate (FPR).

The remainder of the paper is organized as follows. In the next section, existing anomaly and sensor fault detection methods are described in brief. Section 3 presents the proposed sensor anomaly detection method. Experiments and results are discussed in Section 4 along with comparison of the proposed approach against other related approaches. Section 5 and Section 6 provide conclusion and possible future work respectively.

## 2. Related Work

Various approaches have been proposed by researchers to detect anomalies in medical data [23,24,25]. Existing approaches vary from machine learning to data mining. Some notable Machine Learning (ML) approaches are the Naïve Bayes, Bayesian Network and Decision tree methods [26]. The Clustering method in machine learning such as K Nearest Neighbor (K-NN) is used in [22], however this approach is not applicable for standard wireless sensors due to complex computation and high training data storage requirements. Recurrent calculation also demands high energy consumption. Statistical calculation based false alarm detection is proposed in [27], focusing on the Cyber-Physical Systems [28] domain.

Mahalanobis Distance (MD) based approach to detect anomalies are proposed by Liu *et al.* in [29]. The Mahalanobis Distance between predicted and actual multivariate instances is used to detect sensor anomaly. MD considers correlation among multiple attributes. After the arrival of a new instance, MD is calculated between the training data in the sliding window and the current physiological parameter values. If MD is greater than the degree of freedom, abnormal physiological parameters are identified, and the window slides one slot by removing the oldest first instance and adding the new one. The limitation of this method is that it considers that the neighboring sensor nodes collect the same type of data which might not be the case in a healthcare scenario.

Another sensor fault detection system for WSN utilizing piecewise linear models of time series is proposed by Yao *et al.* in [19]. This algorithm is based on the detection of deviation between the reference and the measured time series by using a predefined threshold, and has been evaluated on three types of faults: short time, long time and constant fault. This approach has not been tested on healthcare dataset and prone to high false positive rate due to lack of attribute correlation which makes the method unsuitable.

Linear SVM is used by Salem *et al.* in [17] to detect abnormal instances and linear regression is used for prediction purposes. Linear Regression is a statistical modeling method used to predict the current value of the monitored parameters [30]. Authors claim that SVM ensures computation complexity reduction as the classification is based on the sign comparison of classification and prevents the estimation of each instance on the base station. Linear Regression models a dependent variable *y_ik_* using a vector of independent variables *x_ik_* called regressors. The model is represented by *y_ik_ = C_0_ + C_1_x_i1_ + C_2_x_i2_ + ··· + C_n_x_in_*; where *i* is the instance and C_n_ is the coefficient of the regressors (weights). The sliding window is not used for updating the training data which may reduce the complexity. However, this is a drawback that affects the robustness of the system because of inefficiency in the data update process. Another drawback is that linear regression is not an efficient prediction tool for healthcare application where the physiological parameters have rapid trend change.

Salem *et al.* have developed another method in [16] utilizing Decision tree J48 [31] for classification and outlier detection. Linear regression is used as a prediction tool. In J48 decision tree algorithm, monitored physiological attributes are represented by tree nodes and classes are represented by the leaf nodes. If more than one attribute value defers from the estimated value, an alarm is triggered. Otherwise, the reading is classified as faulty. This method also possesses the drawbacks of not using the sliding window.

From the system model point of view, a brief review of some relevant anomaly detection techniques is presented in this paragraph. An unsupervised distance based anomaly detection technique is proposed by Xie *et al.* in [32] that reduces the dimension of data before distance measurement. The effectiveness of this technique has not been validated for dynamic datasets and is also vulnerable if the dimension is not reduced for multivariate datasets. A clustering method based anomaly detection technique in WSN is proposed by Rajasegarar *et al.* in [33]. Data is clustered and processed before transmitting to the base station. In this distributed reference model based technique each node builds a local reference model and sends it to the based station where a global reference model is built for anomaly detection. The anomaly detection accuracy of this approach is similar to the centralized model, however, its accuracy for online anomaly detection has not been addressed. Many techniques or methods are used for anomaly detection. These techniques differ according to type, whether analysis of data is univariate or multivariate, spatial or temporal reciprocity, if the system is online or offline, its adaptability and data processing location. For a summary see Table 1. Bahrepour *et al.* in [34] propose a decision tree based anomaly detection technique for distributed event detection. Although the time complexity of the proposed technique has been addressed the role of communication overhead for energy consumption has not been addressed. Aggarwal *et al.* in [35] propose a statistical method based anomaly detection technique that is offline, non-adaptive and is a local processing model; whereas Xie *et al.* in [36] propose another statistical method based anomaly detection technique which is online, adaptive and is a distributed model. Both techniques are for univariate data and not effective for multivariate correlated datasets. For further details on the review of data anomaly detection in Wireless Sensor Networks see the work of Rassam *et al.* in [37].

**Table 1:** Summary of Anomaly Detection Techniques.

Type	Technique	Data Analysis	Reciprocity	Online/Offline	Adaptability	Data Processing
Distance/Density-based	MD [29]	Multivariate	Spatial	Offline	Non-adjustable	Central
	k-NN [38]	Univariate	Spatial	Offline	Non-adjustable	Local
	Xie *et al.* [32]	Multivariate	-	Offline	Non-adjustable	Distributed
Clustering Algorithm	Rajasegarar *et al.* [33]	Multivariate	Temporal	Offline	Non-adjustable	Distributed
	Moshtaghi *et al.* [39]	Multivariate	-	Offline	Non-adjustable	Distributed
Decision Tree	J48 [31]	Multivariate	-	Offline	Non-adjustable	Central
	Bahrepour [34]	Multivariate	-	Offline	Non-adjustable	Distributed
Classificatio-based	SVM [40]	Multivariate	Temporal	Online	Non-adjustable	Local
	Linear SVM [15]	Multivariate	Spatio-Temporal	Online	Non-adjustable	Central
Statistical method	Aggarwal *et al.* [35]	Univariate	-	Offline	Non-adjustable	Local
	Xie *et al.* [36]	Univariate	-	Online	Adjustable	Distributed

To address the limitations of the existing sensor anomaly detection methods, we propose dynamic threshold based error computation to detect anomalies in physiological data obtained from each type of sensor and then correlate it with other physiological parameters to differentiate between true and false alarms more effectively. The proposed technique does not utilize any distance and classification based measures for anomaly detection and therefore eliminates the major computational complexity associated with calculating distance and classification. This technique deploys an effective prediction approach and majority voting to achieve higher efficiency in anomaly detection.

## 3. Sensor Anomaly Detection Approach

A remote medical scenario is considered here, where a number of sensors are attached wirelessly to the subjects. In this scenario, N sensors (*S_1_*, *S_2_*, *…*, *S_N_*) collect physiological parameters from the subjects and transmit collected data to the base station or higher capability nodes for processing. Higher capability nodes can be utilized to store sensed data for longer duration for future use. The medical sensors monitor a subject’s condition by collecting and processing various physiological parameters such as Blood Pressure (BP), Heart Rate (HR), Pulse, Respiration Rate, Oxygen Saturation (SpO_2_). For a given time instant *t,* the collected physiological parameters can be denoted as *A_t_ = (a_t,1_*, *a_t,2_*,*…*, *a_t,n_)*, where *n* is the total number of physiological parameters collected by *N* sensors, where *n* ≥ *N*.

**Figure 1:**
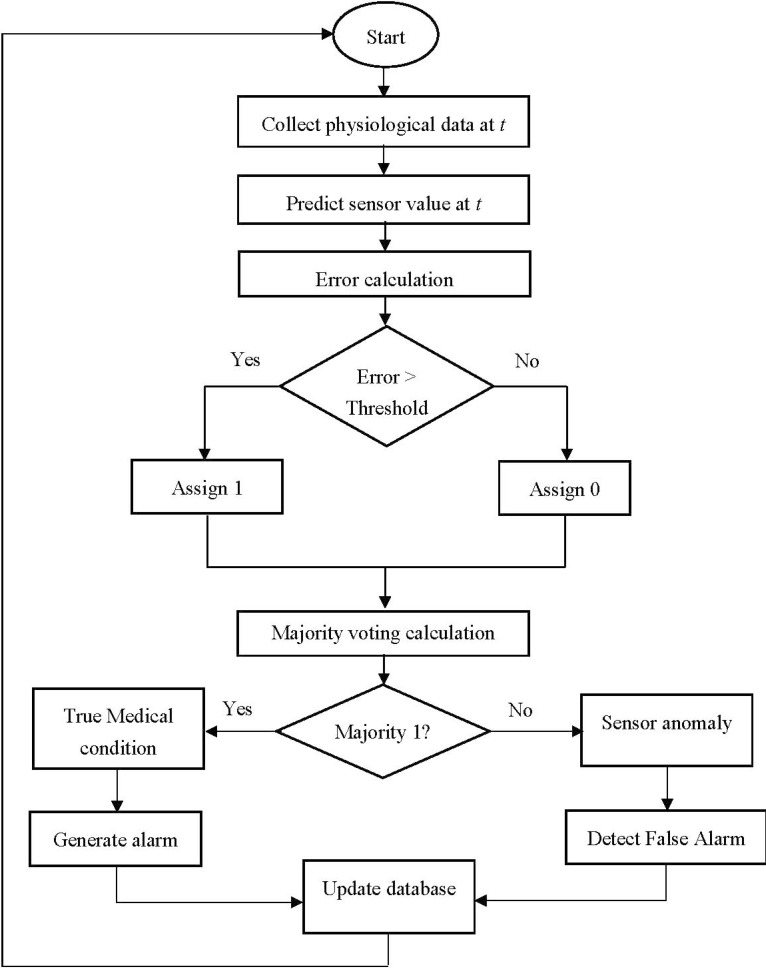
Workflow of the anomaly detection process

As stated previously, the collected data may be unreliable due to sensor malfunction or resource constraint of the sensors. Other possibilities for sensor data anomaly include communication interruption and disconnection from the body. Although it is fairly straightforward to identify the sensors that do not work at all, to identify the sensors that work but sense inaccurate and misleading data is a challenging task. This can cause false alarm or no alarm at all when true medical attention may be warranted. It is important to differentiate false alarms from real alarms and this can be addressed if any sensor anomaly can be detected in real-time with high accuracy [41]. Traditional medical wireless sensor nodes have resource constraints such as power and memory limitation. Their energy levels may deplete rapidly if they are used for the computations required for sensor anomaly detection. To alleviate such constraints, we propose to introduce special nodes called storage nodes [42] to perform the processing required for sensor anomaly detection. A storage node acts like normal sensor nodes in that it performs the basic functions of normal sensor nodes; however, it has higher storage capacity and processing capability to use prediction and outlier detection for detecting sensor anomaly. The storage nodes can be connected to mains power and used as cluster heads [43], so individual sensors in a cluster communicate with the base station via its cluster head [44].

The proposed approach is based on three algorithms: Sequential Minimal Optimization Regression (SMO regression) for the prediction of sensor value, Dynamic Threshold (DT) calculation for error computation, and Majority Voting (MV) for decision on whether to generate alarm. Figure 1 shows the workflow of the anomaly detection process incorporating these three algorithms. SMO regression is used to predict a sensor value at a particular time instant based on the historical data and DT calculation algorithm is used to detect the anomaly. The rationale for using SMO regression in the proposed approach is given in the next section. Finally, MV is used to detect false alarms and true medical conditions. The error calculation step identifies whether the difference between the sensed and the predicted sensor values is higher than a threshold value. Distinguishing false alarm from true alarm is based on whether this threshold is exceeded. Statistical analysis is used on the historical data to determine an accurate threshold value, which is dynamically adjusted with time as new valid sensor data become available.

### 3.1. Selection of Appropriate Prediction Method

Although different prediction methods such as Regression and Gaussian process are widely used as efficient tools for applications such as environment and weather monitoring [45,46]; the healthcare sector is yet to fully utilize prediction as a tool because of its potential high risk impact on *subject/patient* care. We utilize SMO regression to predict sensor values and use the predicted values to detect anomalies and potential data anomaly. This is expected to reduce false alarms and thereby improve system efficiency. The predicted value is used for comparison purposes only. In addition, prediction system failure is a rare event because historic data are always available in the system. 30 samples worth of past data is sufficient to successfully build the SMO regression prediction model which is reasonably small and hence acceptable amount of time in patient monitoring scenario. As various prediction methods reported in literature are not particularly developed for healthcare, it is important to identify the prediction methods that are most suitable for healthcare, because in these applications it is essential to have the ability to build models fast with high accuracy and the ability to adapt quickly to rapidly changing trends in datasets.

**Figure 2:**
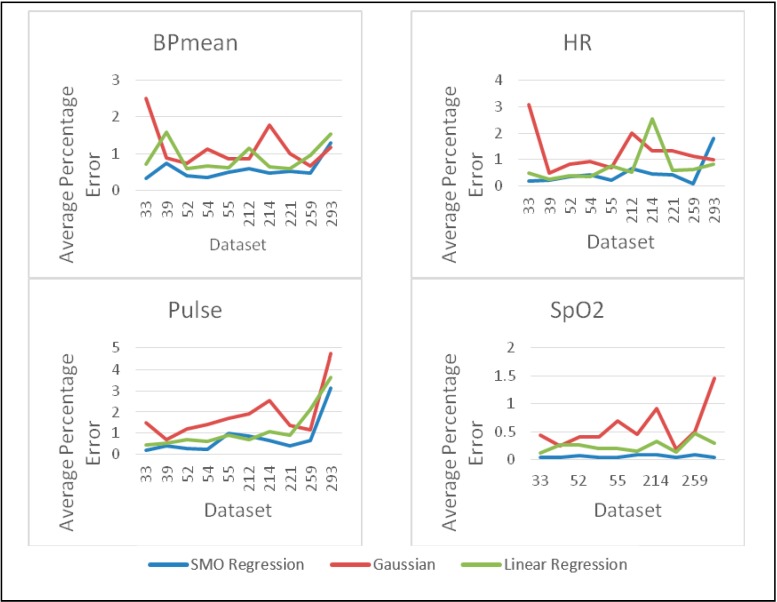
Comparison of prediction methods for average percentage error calculation.

**Figure 3:**
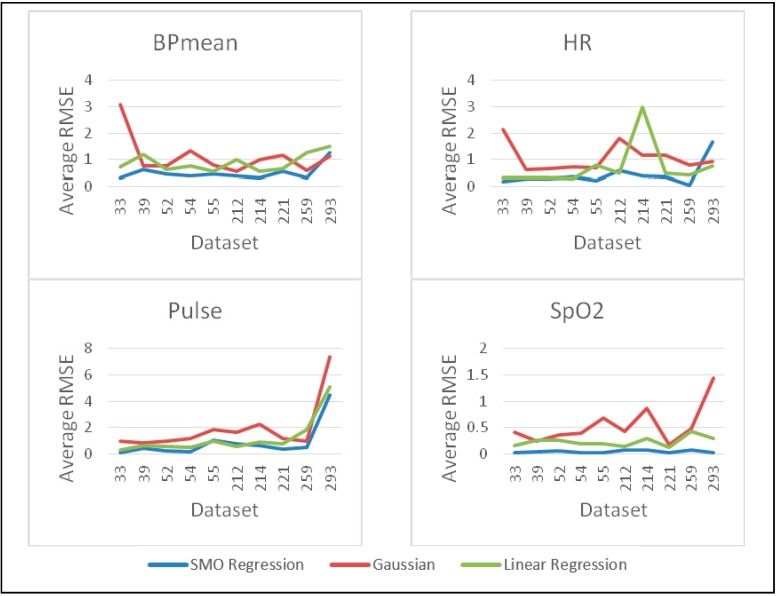
Comparison of prediction methods for average RMSE calculation.

To identify the most suitable prediction method for healthcare, we have compared three established prediction methods (Linear Regression, Gaussian Process and SMO Regression) on 10 real healthcare datasets [47]. Each dataset contains more than 80,000 data samples of physiological parameters, namely BP mean, HR, Pulse, Respiration and SpO_2_. More than 30 runs are performed to collect 100 predicted data from each dataset and then compared with real data from Physionet database [47] to measure Root Mean Square Error (RMSE) and percentage error. 30 samples are taken as past data to build the prediction model. The average percentage error and average RMSE for all 10 datasets are given in Figure 2 and Figure 3 for all the three methods. Clearly, for all datasets, the SMO regression based prediction method has performed better than the other two methods.

### 3.2. Prediction Model

We use Sequential Minimal Optimization (SMO) Regression algorithm to build our prediction model. A brief discussion on the prediction model is given below:

SMO Regression [48] is an extension of Sequential Minimal Optimization (SMO) algorithm [49]. Suppose we have training data [(*x_1_, y_1_*), ..., (*x_l_, y_l_*)]⊂ *χ x ℝ*, where *χ* denotes the space of the input patterns (for example, *χ = ℝ^d^*). Which can be the past medical data collected from the medical sensors. A function *f(x)* with most error (*ε*) deviation from the actual training data. The errors are to be neglected as long as the values are less than ε. This is crucial as losing more than ε will deteriorate the system performance when dealing with medical data. For linear functions *f*, taking the form [48,49]:

(1) f(x)=〈w,w〉+b with w∈χ, b∈ℝ

where <· , ·> denotes the dot product in *X*. Equation (1) means that it looks for a small *w* which can be ensured by minimizing the norm, *i.e.*, ‖*w*‖^2^ = ‹*w*,*w*›. This is a convex optimization problem [48]:

$$\begin{array}{l} \text{minimize}\;\frac{1}{2}{\left\|w\right\|}^{2}\\ \text{subject}\;\text{to} \end{array}$$

(2) $$\begin{array}{c} y_{i}-\langle w,w\rangle -b\leq \varepsilon \\ \langle w,w\rangle +b-y_{i}\leq \varepsilon \end{array}$$

The slack variables ξ_i_, ξ_i_^*^ are introduced to deal with the optimization problem stated in Equation (2). This leads to the formulation stated in Equation (3) as in [48]:

$$\begin{array}{l} \text{minimize}\;\frac{1}{2}{\left\|w\right\|}^{2}+C\sum_{i=1}^{l}\left(\xi_{i},\xi_{i}^{*}\right)\\ \text{subject}\;\text{to} \end{array}$$

(3) $$\{\begin{array}{c} y_{i}-\langle w,x_{i}\rangle -b\leq \varepsilon \\ \langle w,x_{i}\rangle +b-y_{i}\leq \varepsilon \\ \xi_{i},\xi_{i}^{*}\geq 0 \end{array}$$

*C* > 0 is a constant that determines error range.

The experimental results presented in Figure 2 and Figure 3 demonstrate that SMO regression provides better prediction accuracy compared to other available methods, namely Gaussian and Linear Regression. Therefore, sensor anomaly detection system proposed in this paper aims to benefit from the enhanced prediction capability of SMO regression. To the best of our knowledge, for medical wireless sensor networks, SMO regression has not been used previously for prediction in sensor data anomaly detection system.

### 3.3. Dynamic Threshold Based Error calculation

As soon as the storage node or sink node receives the sensor data of physiological parameters, prediction for the next sensor value starts using the past values. As storage nodes [42] are special nodes with higher storage and processing capability, the storage and processing cost do not degrade the overall network due to energy consumption. The storage node eventually receives the actual sensor data which is compared with the predicted data. Error is calculated as the difference between the sensed and the predicted sensor values. If the error for a particular parameter is less than the threshold value for that parameter then the predicted value is updated by the actual sensed value. The system then prepares for the next prediction. The threshold value for every parameter under consideration is determined by statistical analysis on the subject’s *past/historic* data. In case the error for a particular physiological parameter is greater than the threshold value, that particular parameter is correlated with other physiological parameters in the Majority Voting stage described in Section 3.4. For most medical conditions a number of parameters such as BP, Pulse, HR, Respiration and SpO_2_ vary in a correlated manner. Therefore, it is possible to determine whether the abnormal value of a parameter is justified based on the values of other related physiological parameters. If not, the collected parameter will be identified as anomalous and any possible alarm will be declared as false alarm.

#### 3.3.1. Analysis of Dynamic Threshold

The threshold value for any physiological parameter may vary from one subject to another depending on factors such as age, physiological condition and life style. The threshold value may vary even for the same subject due to variations in physiological condition. Therefore, a fixed threshold value will fail to calculate error accurately for a subject in different instances of time. Thus, it is important to have the ability to adjust the threshold value to one that reflects the actual overall physiological condition of the subject. Such a *dynamic threshold* value can be determined based on the subject’s immediate past physiological data at the same time when prediction of a sensor value is performed based on the same historic dataset. Standard deviation of a certain window of historic data allows an upper and lower bound to be used as a local anomaly score. Incrementally updating the window with time provides a contextual viewpoint of the data that makes the threshold value dynamic and makes it more relevant to the data at various points in time. This dynamic threshold value is updated over time throughout the experiment.

Let *x* = (*x_1_*, *x_2_*, *…*, *x_n_*) be an array of immediate past physiological parameters. A dynamic threshold value, denoted as *T_d_*, is obtained for each physiological parameter (say *x_1_*) by calculating the standard deviation of the individual physiological parameters of *x*. As stated above, the dynamic threshold (*T_d_*) has advantage over a fixed threshold (*T_f_*), because the former reflects the actual physiological condition of the subject at a particular instance. Let the error in any physiological parameter is *e*, the value of *e* being a fraction. Let the total error of the array *x* be *e*(*T_d_*) when dynamic threshold is used and for fixed threshold the error is *e*(*T_f_*).

**Theorem 1.** *For any x, e*(*T_d_*) ≤ *e*(*T_f_*)

**Proof:** For *n* number of parameters, when using fixed threshold *T_f_*, the number of errors increases as follows:

$$e(T_{f})=\sum_{i=1}^{n}\left(x_{i}+e\right)$$

whereas for dynamic threshold, $e(T_{d})=\sum_{i=1}^{n}(x_{i})+e$. Clearly, *e*(*T_d_*) ≤ *e*(*T_f_*). Therefore, the total error for *n* number of parameters is minimized when dynamic threshold is used.

#### 3.3.2. Statistical Viewpoint of Dynamic Threshold

**Figure 4:**
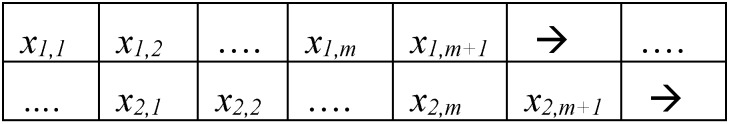
Sliding window for dynamic threshold determination.

Given an array *x*, we now look at how to find a dynamic threshold (*T_d_*) for the parameter *x_1_* based on its historic values at time instants *1*, *2*, …, *t*. As shown in Figure 4, by arranging the historic values, *i.e.*, *x_1,1_*, *x_1,2_*, …, *x_1,t_* and calculating *T_d_* = *S_d_* (*x_1,1_*, *x_1,2_*, …, *x_1,t_*) provides the desired threshold, where *S_d_* is standard deviation. However, for physiological parameters of varying range, this method would end up providing a fixed threshold. To deal with this limitation, we utilize sliding window to calculate dynamic threshold. This provides the proposed technique the ability to adapt with the continuously changing physiological parameters quickly.

The standard deviation (*S_d_*) of the updating array provides dynamic threshold value which changes with the course of subject’s condition and time. This dynamic threshold is utilized to calculate the error of the physiological parameter. Error calculation algorithm is presented in Algorithm 1.

**Algorithm 1** Dynamic Threshold Calculation and Error Computation Algorithm1:**PROCEDURE**: Determine dynamic threshold2:**Input**: *S_a_*, *n;* Actual sensor data: *S_a_* ; *Number of past data: n*4:**Output:**
*S_d_*, *T_h_*; Standard deviation: *S_d_*; Threshold: *T_h_*5:**for**
*S_a_* = 1 : *n*
**do**6:calculate *S_d_;*7:*T_h_ ← S_d_;*8:Return *T_h_ ;*9:Update sliding window;10**End**11:**PROCEDURE**: Error calculation12:**Input**: *S_a_*, *S_p_, t;* Actual sensor data: *S_a_* ; Predicted sensor data: *S_p_; Particular instance: t*13:**Output:**
*e*; Percentage Error: *e*14:**for**
*S_a_ (t)* and *S_p_ (t)*15:calculate *e;*16:Return *e*;17:**End**

### 3.4. Majority Voting

Each subject is associated with a number of different types of sensors that measure different physiological parameters. Majority voting is performed for all the different physiological parameters measured for an individual subject. The measured values of the physiological parameters are compared with the parameter values predicted for the corresponding sensors. Each physiological parameter is assigned a status of *1* or *0* indicating that the parameter is *anomalous* or *normal*. Assignments of all the physiological parameters of a subject are received and forwarded to voting. If the votes are greater than the average number of physiological parameters, the decision is made whether or not the sensor value is faulty based on the majority voting. We present an analysis to show the performance of the majority vote. Assume that the number of sensors is *N* and number of physiological parameters is *n*, where *n* ≥ *N*. Votes from the *n* are denoted as *v*(*1*), *v*(*2*), *…*, *v*(*n*) to assess the status (*true alarm/ false alarm*) of the system. The decision from the sensors is expressed as *v_i_* ϵ (*0*, *1*) that is used for voting. If *Y* is greater than the average of the number of physiological parameters *n*, the majority vote decision is published on *true alarm* or *false alarm*. It will be declared as *true alarm* if the outlier parameters are more than the average of the total number of parameters and it will be false alarm if the outlier parameters are less than the average of the total number of parameters. The algorithm is presented in Algorithm 2.

**Algorithm 2** Outlier Detection Voting1:**PROCEDURE**: Outlier Detection using Voting2:**Input**: *S_a_*, *S_p_*, *n*; Actual sensor data: *S_a_*; Predicted sensor data: *S_p_*; Number of physiological parameters*: n*3:**Output**: *A_t_*, *A_f_*; *True alarm: A_t_; Sensor anomaly = False alarm: A_f_*4:**for**
*i* = 1 : *n*
**do**5:error(e) between *S_a_(i) and S_p_(i) > threshold*6: *y(i) ← e (i)*;7: **if**
*count*(*y* = i) > *average n*
**then**8:  Return *A_t_*;9: **Else**10:  Return *A_f_*;11:**end if**12:**end**

## 4. Experiments and Results

Experiments are conducted on real medical datasets [47] in Java environment to determine and compare the accuracy of the proposed sensor anomaly and *true/false* alarm detection method. For the prediction part, the regression utilities of the WEKA tool [50] are used. In order to assess the performance of the proposed system we utilize physiological parameter data from Multiple Intelligent Monitoring in Intensive Care (MIMIC) database of Physionet [47]. Sensed value of each parameter is compared with the predicted value. Prediction model is constructed using SMO regression method for all physiological parameters. Historic data are used to build the prediction model. The parameters for experimentation are given in Table 2.

**Table 2:** Parameters for experimentation.

Parameter	Value
Sliding window samples for dynamic threshold	30
Sliding window samples for prediction model	30
Anomaly detection window samples	80
Data samples used for prediction model selection	80,000
Number of physiological parameters	5 (ABP, HR, Pulse, Respiration, SpO_2_)

Sensor data anomaly is determined by correlating other physiological parameters. Sliding window is used to update the data array and dynamic threshold is determined using statistical calculation of standard deviation. These dynamic thresholds are used to calculate the error. Then majority voting is performed to detect the true alarm or false alarms. We further compare and evaluate the performance of the proposed approach with Mahalanobis Distance (MD) [29], Linear SVM [17] and J48 [16]. We have used the same datasets (MIMIC DB datasets 221, 052 and 293) for the comparison of our proposed approach against MD, Linear SVM and J48. In Mahalanobis Distance (MD) approach, the distance is measured between monitored attributes. MD follows the degree of freedom which is utilized as threshold for anomaly detection. In linear SVM method, the classification model is built in training phase. In testing phase, the inputs are classified as normal or abnormal based on the classification model. If abnormal values are detected by the SVM then the prediction model is initiated using linear regression. If the Euclidean distance between measured and estimated value is deviated by 10% of the estimated value, the measured value is replaced by the estimated value obtained by linear regression. This method possesses two major drawbacks. Firstly, replacing measured value with an estimated value gained by linear regression may be critical for healthcare scenario where a miscalculated health data can be harmful to patient. In addition, linear regression is no exception to having estimation error. Secondly, this method does not have sliding window which is inefficient for dynamic systems such as healthcare. In the J48 method, a J48 decision tree model is built to classify normal and abnormal data. The tree model is fast and inexpensive to build. If the data is classified as abnormal by J48, it is assumed that the attribute is missing and linear regression is used to estimate the replacement value. If at least two attribute values are exceeding the predefined threshold value (by 10% of estimated value), the alarm is raised for the caregiver’s intervention. Sliding window is not used in this method for updating data. This makes the approach prone to misclassification and estimation errors. This may lead to increased false positive rates. Compared to these methods, our proposed method does not replace any actual value by the estimated value obtained from prediction model, rather the estimated value is used for anomaly detection purpose only. In addition, our proposed method uses sliding window for dynamic threshold measurement. This makes the proposed system suitable and efficient for dynamic systems such as healthcare with potentially enhanced performance compared to the other methods discussed here.

**Figure 5:**
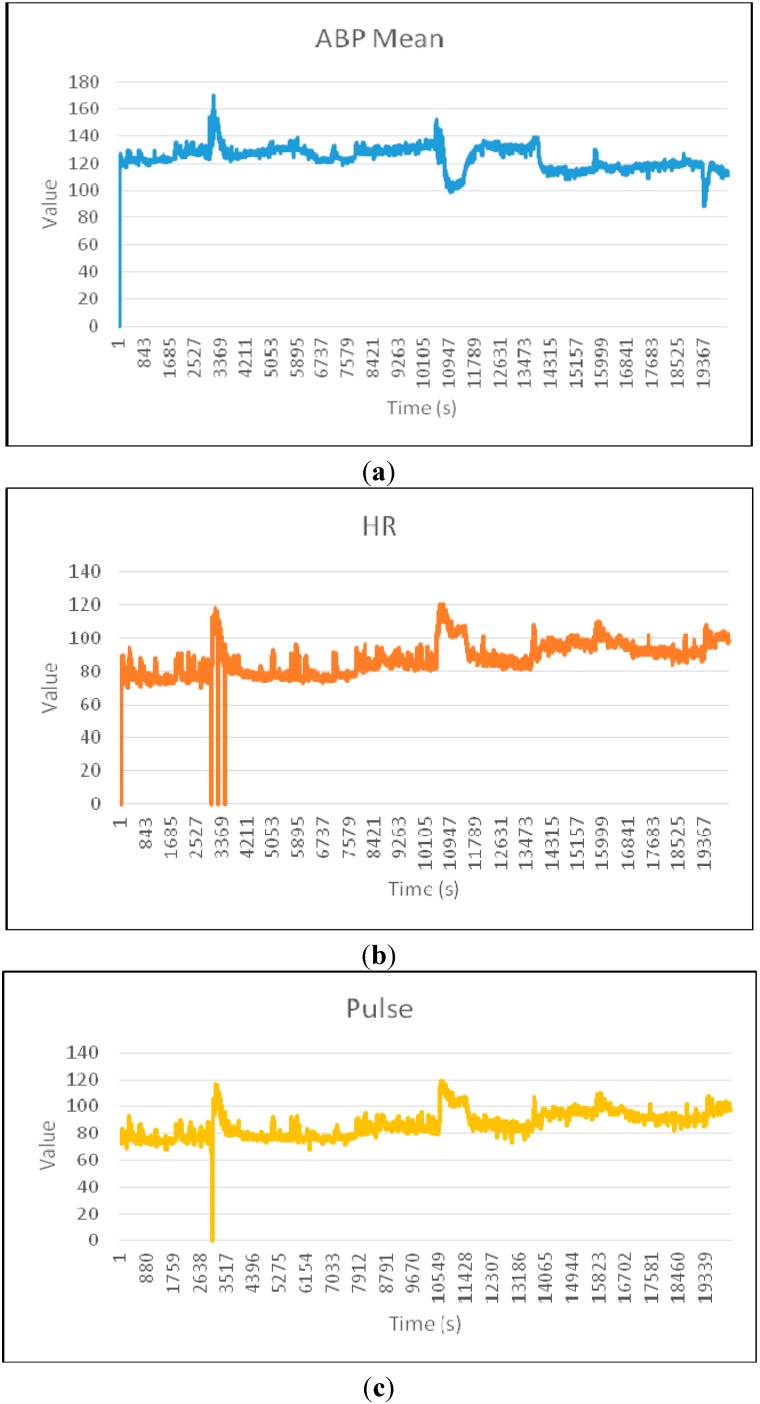

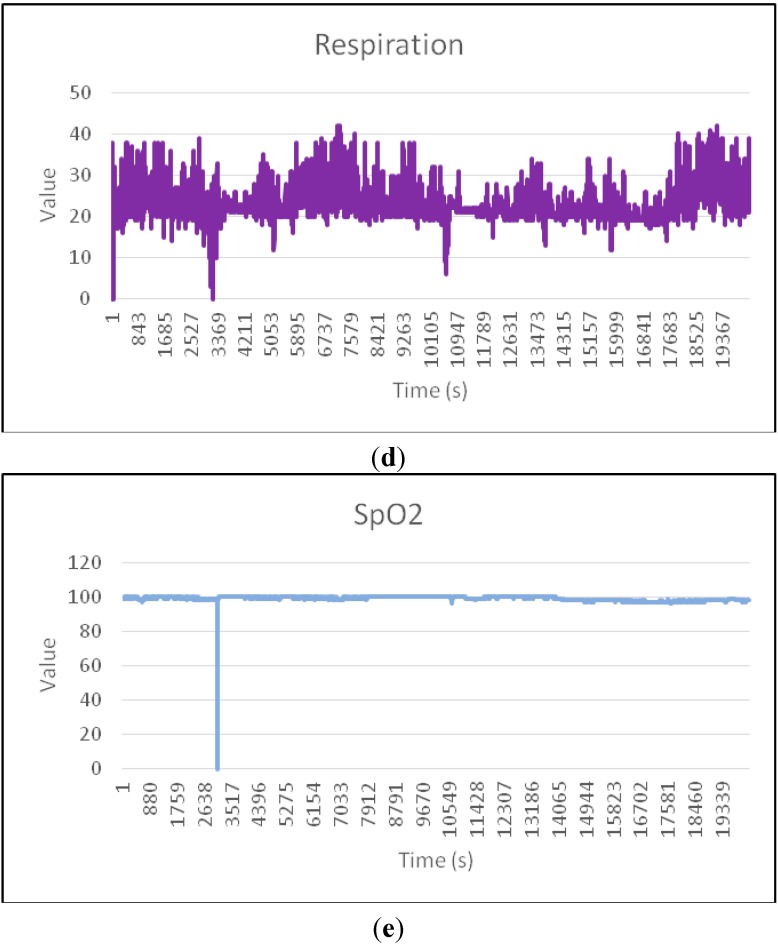
(**a**) Arterial blood Pressure (ABP) mean; (**b**) Heart Rate (HR); (**c**) Pulse; (**d**) Respiration; (**e**) Oxygen Saturation (SpO_2_).

We have used the WEKA software to perform experimentation on SVM and J48. We have used Java for the experimentation on Mahalanobis Distance. For clear representation of the physiological parameters in the experimentation, we present Figure 5a–e to show the variations of the individual physiological parameters of dataset 221.

**Figure 6:**
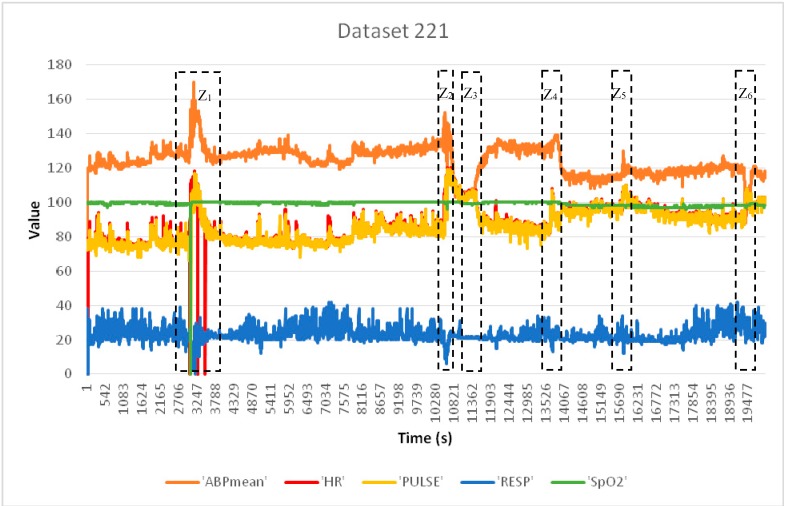
Correlation of Physiological Parameters.

The heart rate is measured in beats per minute (bpm), and the normal values for heart rate are within the interval (60–100) for a healthy adult at rest. The pulse is measured in beats per minute (bpm) and BP is measured in millimeters of mercury (mmHg). Average resting respiration rate for adults is 12 to 20 breaths per minute and normal SpO_2_ rate must be within the range (95%–100%) [51]. In Figure 6, six zones (Z_1_–Z_6_) can be visually identified to have potential anomalies and marked with Z_1_–Z_6_ in dotted boxes. It can be observed from Figure 6 that HR and Pulse show anomaly in box Z_1_ around the 3000th time instance. Usually, the heart rate and pulse must have the same values and must show the same variations, as they represent the same physiological parameter monitored through two different sensor devices. As there are differences in values between HR and Pulse, we mark this point as potential sensor anomaly for HR sensor. However, from Figure 5b,c we observe that the sensed values dropped around 2997 time instance and this incident is supported by other correlated parameters in Figure 5b,d,e. In Figure 5a, Arterial Blood Pressure increased around 2997 instance. Also Respiration and Oxygen saturation drop around 2997 instance in Figure 5d,e, respectively. This incident is marked as true medical condition. Area Z_2_, Z_4_, Z_5_ and Z_6_ of Figure 6 are also correlated regular medical incidents and may not be serious condition, however area Z_3_ presents a sharp drop in BP but no visible change in respiration rate at the same instance. This respiration sensor is representing potential data anomaly. In Figure 6, we notice one abnormal reading with zero values for SpO_2_ followed by normal values around box Z_1_ and a lower value is the sign of asphyxia, lack of oxygen and heart disease [52]. This represents potential severe medical condition and supported by other parameter change. On the other hand if there is no spatial correlation among monitored physiological parameters, it indicates potential sensor fault. Change in a particular sensor value without correlation with other parameters indicates sensor anomaly.

### 4.1. Performance Analysis

To analyze the performance of the proposed approach, we consider the anomalies that are identified as true medical condition. Receiver Operating Characteristic (ROC) curve [53] is used to present the performance of the proposed technique on the Detection Rate and the False Positive Rate. In a two-class prediction problem (binary classification) [53], the system outcomes are labeled as positive outcome (*p*) or negative outcome (*n*) and there can be four possible outcomes from a binary classifier. If the test outcome from a prediction is *p* and the actual value is also *p*, then it is called a True Positive (TP). On the other hand, if the actual value is *n* then it is a False Positive (FP). Similarly, True Negative (TN) is achieved when both the prediction outcome and the actual outcome are *n*, and False Negative (FN) is achieved when the prediction outcome is *n* while the actual value is *p*. The Detection Rate can be calculated as:

(4) $$\mathrm{Detection}\;\mathrm{Rate}=\frac{TP}{TP+FN}$$

where TP is the number of True Positives, and FP is the number of False Positives.

The False Positive Rate (FPR) can be calculated as:

(5) $$\mathrm{False}\;\mathrm{Positive}\;\mathrm{Rate}=\frac{FP}{FP+TN}$$

**Figure 7:**
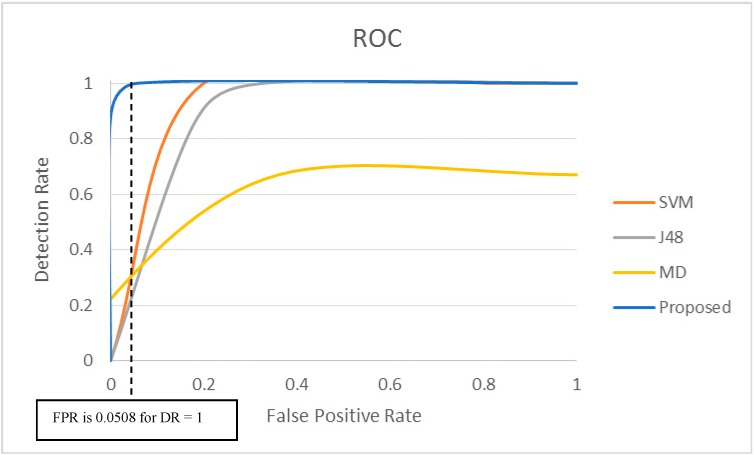
ROC curve for the proposed method for dataset #221 compared with other methods.

The ROC curve presented in Figure 7 shows the relationship between the detection rate (DR) and the false positive rate (FPR) for our proposed approach and related approaches. Intermediate data points in the ROC curve can be achieved by varying the number of physiological parameters and injecting random synthetic anomalies at different time instances to evaluate the detection accuracy. We have changed the number of physiological parameters (*k*) for generating the ROC. Changing the *k* impacts detection rate and false positive rate due to final outcome of majority voting varies with the number of physiological parameters participating in voting. It is not the impact of the dynamic threshold.

Ideally an anomaly detection mechanism should have a high Detection Rate and low False Alarm Rate. First, we have applied the proposed anomaly detection scheme to detect anomalies in the dataset and then applied majority voting flags to determine whether any alarm generated is true or false. Using Equations (4) and (5), the values of Detection Rate (DR) and False Positive Rate (FPR) are calculated for the proposed technique. These values determine the ability for the proposed technique to identify true alarm and false alarm. The ROC curve in Figure 7 clearly shows that our proposed approach achieves a Detection Rate of 100% at a False Positive Rate of 0.0508, *i.e.*, at FPR = 5.08%. FPR value is calculated using Equation (5) where False Positive (FP) is 3 and True Negative (TN) is 56. False Positive (FP) is achieved when actually there are false alarms, however the test system flagged these 3 false alarms as true alarms due to error. True Negative (TN) value is achieved where the actual false alarms are correctly flagged as false alarms in test system. The performance of other related approaches are also analyzed in Java environment over the same medical data sets and the results are presented in Figure 7. The SVM and J48 methods can potentially achieve 100% DR at the cost of higher FPR values of 20% and 33% respectively. The MD approach can only achieve DR of up to 67% and incurs a much higher FPR of 36%. With a Detection Rate of 100% at a False Positive Rate as low as 5.08%, the proposed method clearly outperforms the other three methods and provides much better outcomes for sensor anomaly detection in healthcare. The FPR of 5.08% is acceptable according to the other real world false alarm rates reported in [54].

**Figure 8:**
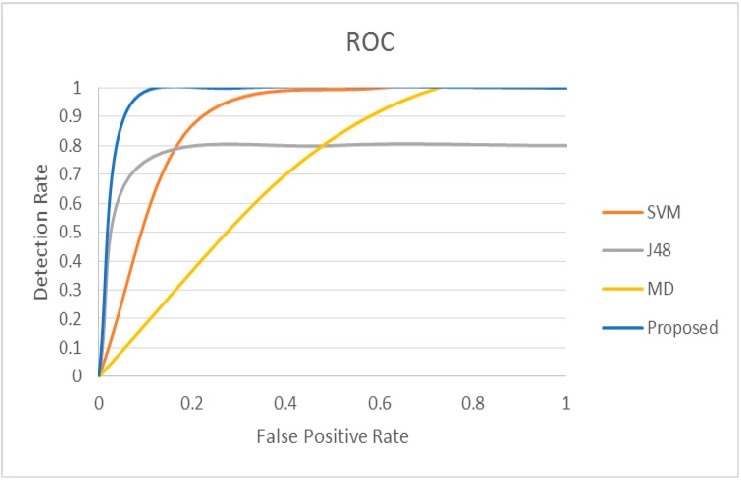
ROC curve for the proposed method for dataset #052 compared with other methods.

**Figure 9:**
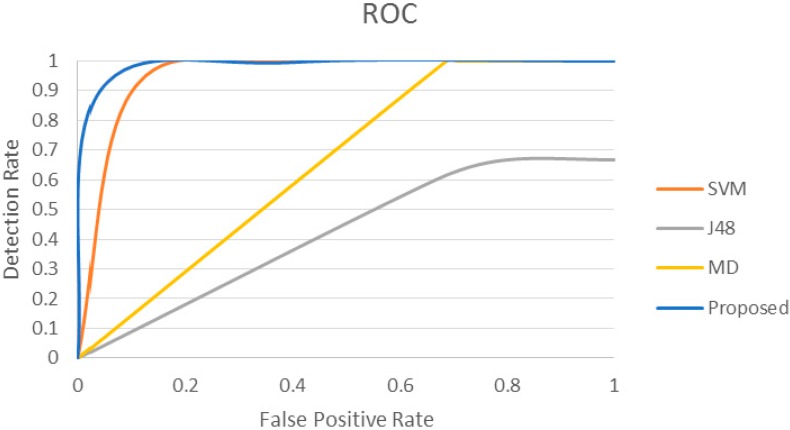
ROC curve for the proposed method for dataset #293 compared with other methods.

We conducted further experiments using two additional datasets (Physionet MIMIC Database Numerics 052 and 293 [47]) and compared the proposed approach with other related methods. Datasets 052 and 293 also contain samples of patients’ physiological parameters such as ABPmean, HR, Pulse, Respiration and SpO_2_. Since the selected datasets do not contain any pre-existing anomaly, synthetic anomalies are inserted randomly in both datasets to investigate the anomaly detection performance of various approaches. Original physiological parameters are replaced by the synthetic anomalies that are generated by changing the original data values by 50%, 30% and 20%. This approach ensures the presence of false alarms and true alarms in the dataset for evaluation purpose. Experimental results are presented in Figure 8 and Figure 9. The proposed approach presents DR = 100% and FPR = 9.091% for dataset 052, whereas DR = 100% and FPR = 16.7% for dataset 293.

Unlike the experimental results presented in Figure 7 for dataset 221, J48 presents detection rates of 80% and 66.7% for datasets 052 and 293 respectively (Figure 8 and Figure 9) due to misclassification of True Alarms as False Alarms. In addition, some anomalous data values for True Alarms are close to the normal parameter values. J48 classifies them as False Alarms, which leads to classification errors. On the other hand, MD and SVM approaches present 100% detection rate for dataset 052, however the False Positive Rates are 72.73% and 40% respectively (Figure 8). For dataset 293 (Figure 9), MD and SVM approaches also present 100% detection rate, whereas the False Positive Rates are 68.75% and 20% respectively. In both cases the False Positive Rates are much higher than the proposed approach.

From the results presented on three medical datasets (221, 052 and 293), it is clear that the sensor anomaly detection approach introduced in this paper presents 100% detection rate for all three datasets and much lower false positive rates for all datasets compared to the other approaches. Therefore the proposed approach exhibits superiority over the other related approaches in all cases by scoring high DR and lower FPR.

For a window of 50 data samples, each containing 5 physiological parameters, anomaly detection calculations with majority voting are performed within 9.455 s. Thus the complete response time is 0.1891 s for each sample. The experiment is performed on a PC with an Intel core i3, 2.3 GHz processor and 4 GB RAM.

## 5. Conclusions

In this paper, a sensor anomaly detection system to distinguish true alarms from false alarms has been presented for healthcare applications. The proposed system predicts a sensor value based on historic data and compares it with the actual sensed value. Then dynamic threshold is utilized to calculate the error followed by majority voting to identify true alarm or false alarm. The proposed approach has been implemented in Java environment, leveraging the SMO regression utility of the WEKA tool. The implemented system is tested through experiments conducted on real medical datasets and compared with existing approaches. The experimental results have demonstrated the effectiveness of the proposed system, presenting a high Detection Rate (DR) of 100% for all three medical datasets, and lower False Positive Rates (FPR) for all the datasets. The high Detection Rate and lower False Positive Rates make the proposed system very competitive compared to other systems reported to date.

## 6. Future Work

This work can be further enhanced using machine learning based dynamic threshold detection and weighted correlation based feature selection for multivariate data. A Machine Learning based dynamic threshold detection technique may help determine contextual anomalies for physiological parameters more precisely. Another potential benefit of this approach will be to adequately deal with the uniqueness of individual’s physiological parameters. Utilizing weighted correlation will potentially improve the system performance further in online processing, where the important features will be first identified and more focus will be placed on the parameters based on their importance.
